# Monthly Botanical Report

**Published:** 1812-04

**Authors:** 


### MONTHLY BOTANICAL REPORT.

SINCE our last Report, we have received another number of the BOTANIST'S REPOSITORY, the contents of which we shall briefly notice.

PROTEA *radiata*. A very shewy species, which flowered at Mr. Knight's, King's-road, Little Chelsea. It seems to be nearly allied to the protea *coronata* of the same work, which is the *formosa* of Salisbury and Brown. Mr. Andrews describes the leaves as "standing sideways in a horizontal direction." Linnæus has defined a horizontal leaf as a position that forms a right angle with the stem, and a vertical one as standing quite upright, or forming a right angle with the horizon; at least, this is Willdenow's explanation in his Principles of Botany. But, in practice, he seems to have used these words differently, meaning by the first a leaf in the most natural position, having the upper surface looking towards the sky, the under one towards the earth; by the latter, or a vertical leaf, one that has its base so twisted as to bring one of its margins upwards, the other downwards, and the two surfaces to the horizon. It is in this sense that these words are applied in his distinguishing characters of the species of Lactuca, where he describes one species as having *folia verticalia*, another *folia horizontalia*. Dr. Smith's definition of *vertical* corresponds with the latter sense.

ANDROSACE *coronopifolia*. Seeing that the *caryophylleæ* are so subject to vary in respect to size, we should be inclined to think that this might be a mere variety of Androsace *lactea*, under which name Mr. Bell received it from Siberia; but the serrature and depressed growth of the leaves, as represented in this figure, certainly lead to a different conclusion.

CHOTOLARIA *saltiana*. Supposed to be a new species, with yellow flowers, from Abyssinia, whence the seeds were brought by Mr. Salt, and raised in Mr. Lambert's stove at Boyton.

ALTRŒMERIA *edulis*. Said to be hardly distinguishable from A. *Salsilla*, except by the roots, which in the latter are woody and long; whereas, in this plant, they are round and succulent, the size of young potatoes, and contain a farinaceous substance, from which is prepared a wholesome and agreeable mucilage, called here cream; but jellies, or more properly mucilages, made of starch or flour, are not usually so termed in English. The Negroes are said to seek eagerly after this plant, not for the sake of its beautiful flowers, but of its roots, which not only serve them for food, but as an article of commerce; selling them under the name of white Jerusalem artichokes, at Cape Francois, as must be meant; but, from a most ridiculous blunder, it is here said that for the above purpose "they take them with them when they travel to the Cape of Good Hope." But with our author distance is nought; and time past and present are as one; for he adds, that "all the species which compose this fine genus *are* to be found in the superb gardens of the Incas of Peru, where it is indigenous!!!"

XERANTHEMUM *fasciculatum*; a red-flowered variety. We suspect, however, this may be a distinct species, but the other varieties

 published published in the Botanist's Repository belong to Xeranthemum *sessamoides* of Linnæus, as has been long ago observed in the Botanical Magazine. Willdenow has nevertheless made them distinct, having both Elichrysum *sessamoides*, purple and white varieties, and the same of Elichrysum *fasciculatum*, in which he undoubtedly errs.

The Baron Jos. Francis Jacquin, son of the celebrated botanist, has published a decade of rare plants, with colored figures, in folio, much in the style of his father's Hortus Vindobonensis. The plants figured in this fasciculus are:

1. VALLISNERIA *spiralis*.

2. SCHOLLIA *crassifolia*. Asclepias *carnosa* of Willdenow, Botan. Magazine, Exotic Botany, and other authors. Mr. Brown has been beforehand with him in assigning the name of HOYEA to this genus.

3. SALVIA *scabiosæfolia*. S. *hablitziana* of the Botanical Magazine and Willdenow.

4. ACHILLEA *tenuifolia*. Willd.

5. CARLOWIZIA *salicifolia*. *Carthamus salicifolius*. Willd.

6. SALANUM *fastigiatum*. Willd. Hort. Berol.

7. ——— *brancæfolium*. S. *decurrens*. Balbis stirp. Hort. acad. Taurin.

OXALIS *tetraphylla*. Willd.

ÆSCHULUS *macrostachya*. Michaux, Willd.

10. CENTAUREA *Balsamita*. Willd.

In the Botanical Report, which occasionally indulges in criticism, it may not be thought altogether impertinent to take some notice of a letter which appeared in the Monthly Magazine for last month, signed James Hall, in which the writer observes, that, not long ago, he saw some people at Walthamstow gathering mandrakes. These, he says, are an innocent species of night-shade, called Atropa by the botanists, and that it is often called briny (briony) by the common people. It is difficult to conceive so many errors to be accumulated in so short a space as occur in these few words; which, however, would not be worth noticing, except that some of them are of a nature that might lead to hazardous experiments. In the first place we must observe, that the mandrake (Atropa *Mandragora*) is not indigenous to Great Britain, and that it is by no means an innocent species of night-shade, being a drastic purgative. There is not, however, much reason to believe that the mandrakes found by Leah's son had any thing to do with this plant, which probably got its name, and supposed aphrodisiacal virtues, from the circumstance of its root being sometimes divided into two straight tap roots, assuming thus some likeness to the form of a man, a resemblance which has been frequently aided by art, in order to deceive the curious. The briony is quite a different plant, and common enough in our hedges, and its roots often grow to an immense size, and are variously shaped. Formerly mountebanks, and other deceptive persons, used to cut these roots into various monstrous similitudes, and drying them a few days in sand, to conceal the want of the outer skin, which might betray the fraud, sold them for mandrakes, and before the cheat became too stale to go down, few virtuoso collections wanted one or more of these marvellous roots. At present, the knowledge knowledge of this charlatanery may begin to wear out, and the people whom Mr. Hall saw gathering mandrakes, as they probably called them to him, may have intended to renew an old trick; or a demand may have arisen in the market for these roots, in their natural unsophisticated form, for the purpose of preparing some quack medicine, perhaps a spurious, or, for aught we know, the genuine, preparation of the celebrated remedy for the gout, the Eau Medicinale d'Husson; the briony root possessing qualities not dissimilar to this active medicine, being a powerful narcotic and drastic purgative, highly offensive to the stomach when given in too large doses, and has been formerly particularly recommended in the gout.

Should any of the readers of the Monthly Magazine, trusting to Mr. Hall's account of its being an innocent nightshade, feel inclined to follow the example of the Emperor Julian, and drink the juice of the briony root, Mr. Hall's mandrakes, we beg leave to forewarn him that he will infallibly excite propensities very different from those intended.

What the Dudaim of the Hebrews really were is now totally unknown. Our translation calls them mandrakes, but neither can we now arrive at any certainty respecting the Mandragora of the ancients. The most probable conjecture appears to us that the Dudaim was some kind of eatable fruit.

